# Predicting the indication for adjuvant radiation therapy according to EAU guidelines among patients with high-risk prostate cancer: a novel multivariable model

**DOI:** 10.1038/s41391-025-01018-y

**Published:** 2025-09-03

**Authors:** Carolin Siech, Helge von Kriegstein, Mike Wenzel, Cristina Cano Garcia, Quynh Chi Le, Pierre Tennstedt, Felix Preisser, Tobias Maurer, Maximilian Kriegmair, Felix K. H. Chun, Markus Graefen, Derya Tilki, Philipp Mandel

**Affiliations:** 1 Goethe University Frankfurt, University Hospital, Department of Urology, Frankfurt am Main, Germany; 2 Martini-Klinik Prostate Cancer Center, University Hospital Hamburg-Eppendorf, Hamburg, Germany; 3 Department of Urology, University Medical Center Mannheim, University of Heidelberg, Heidelberg, Germany; 4 Department of Urology Planegg, Munich, Germany; 5 Department of Urology, University Hospital Hamburg-Eppendorf, Hamburg, Germany; 6 Department of Urology, Koc University Hospital, Istanbul, Turkey

**Keywords:** Cancer therapy, Outcomes research

## Abstract

**Background:**

To develop a novel model for preoperatively predicting the indication for adjuvant radiation therapy after radical prostatectomy according to current guideline recommendations of the European Association of Urology (EAU) based on patient and clinical tumor characteristics in high-risk prostate cancer patients.

**Methods:**

Within a high-volume center database (01/2010-08/2024), we identified high-risk prostate cancer patients. Univariable logistic regression models addressed indication for adjuvant radiation therapy. Multivariable logistic regression models included the most informative, statistically significant preoperative predictors. Harrell’s concordance index (c-index) quantified accuracy after 2000 bootstrap resamples for internal validation.

**Results:**

Of 5691 patients, 2137 (38%) had indication for adjuvant radiation therapy according to current EAU guidelines (2025). Indication for adjuvant radiation therapy was associated with higher prostate volume (> 45 cm^3^ and 25–45 cm^3^) and advanced tumor characteristics, namely higher prostate-specific antigen value (>20 ng/ml and 10-20 ng/ml), advanced clinical tumor stage (cT3/4 and cT2), lower number of sampled biopsy cores (≤ 12), higher proportion of positive cores (continuous), and higher Gleason Grade Group in biopsy (5, 4, and 3). No association was observed for age and body-mass index and indication for adjuvant radiation therapy. Multivariable model c-index for the prediction of the indication for adjuvant radiation therapy was 0.761 (95% confidence interval 0.749–0.776).

**Conclusions:**

Clinical tumor characteristics can be used for preoperatively predicting the indication for adjuvant radiation therapy after radical prostatectomy according to current EAU guideline recommendations in high-risk prostate cancer patients. Prior to clinical application, the present multivariable model should be externally validated within an independent cohort.

## Introduction

Radical prostatectomy represents a well-established curative treatment option in patients with non-metastatic prostate cancer [1, 2]. However, selected patients will require additional adjuvant radiation therapy [3–8]. While the treatment guideline recommendations of the American Urological Association (AUA) and the National Comprehensive Cancer Network (NCCN) are cautious regarding adjuvant therapy after radical prostatectomy [9, 10], current prostate cancer treatment guidelines of the European Association of Urology (EAU) recommend adjuvant radiation therapy for patients with pathological negative lymph nodes (pN0) with Gleason Grade Group 4-5 and non-organ confined pathological tumor stage (pT3) with or without positive surgical margins [7, 8]. Moreover, adjuvant radiation therapy is offered to patients with pathological positive lymph nodes (pN1) [7]. These patients with adverse pathology have been shown to benefit from adjuvant radiation therapy [5, 6]. Although all patients are preoperatively informed about the potential risk of adjuvant therapies, it may be difficult to quantify this risk. Moreover, patients may feel psychosocial distress and possibly regret their therapy decision after receiving unfavorable results of their radical prostatectomy specimen and therefore also may benefit from a preoperative improved risk assessment [11–13]. Specifically, Shakespeare et al. stated that men treated with both radical prostatectomy and post-prostatectomy radiation therapy 4-times more often regretted the radical prostatectomy treatment component compared to radiation therapy, which also may come from the uncertainty of the necessity of a radiation therapy [13]. Therefore, for preoperative patient counseling, it may be useful to rely on a model that predicts the risk of adjuvant radiation therapy after radical prostatectomy with high accuracy based on variables that are known prior to curative treatment decision making. As patients with D’Amico high-risk prostate cancer are at highest risk for a multimodal treatment, this patient cohort may benefit most from such model.

We addressed this need and quantified the risk of adjuvant radiation therapy according to current EAU guideline recommendations by relying on a contemporary cohort of patients with D’Amico high-risk prostate cancer treated with radical prostatectomy in a high-volume center. Furthermore, we postulated that a multivariable model, relying on preoperative known patient and clinical tumor characteristics, may predict the indication for adjuvant radiation therapy after radical prostatectomy according to current EAU guideline recommendations.

## Materials and methods

### Study population

**Fig. 1:**
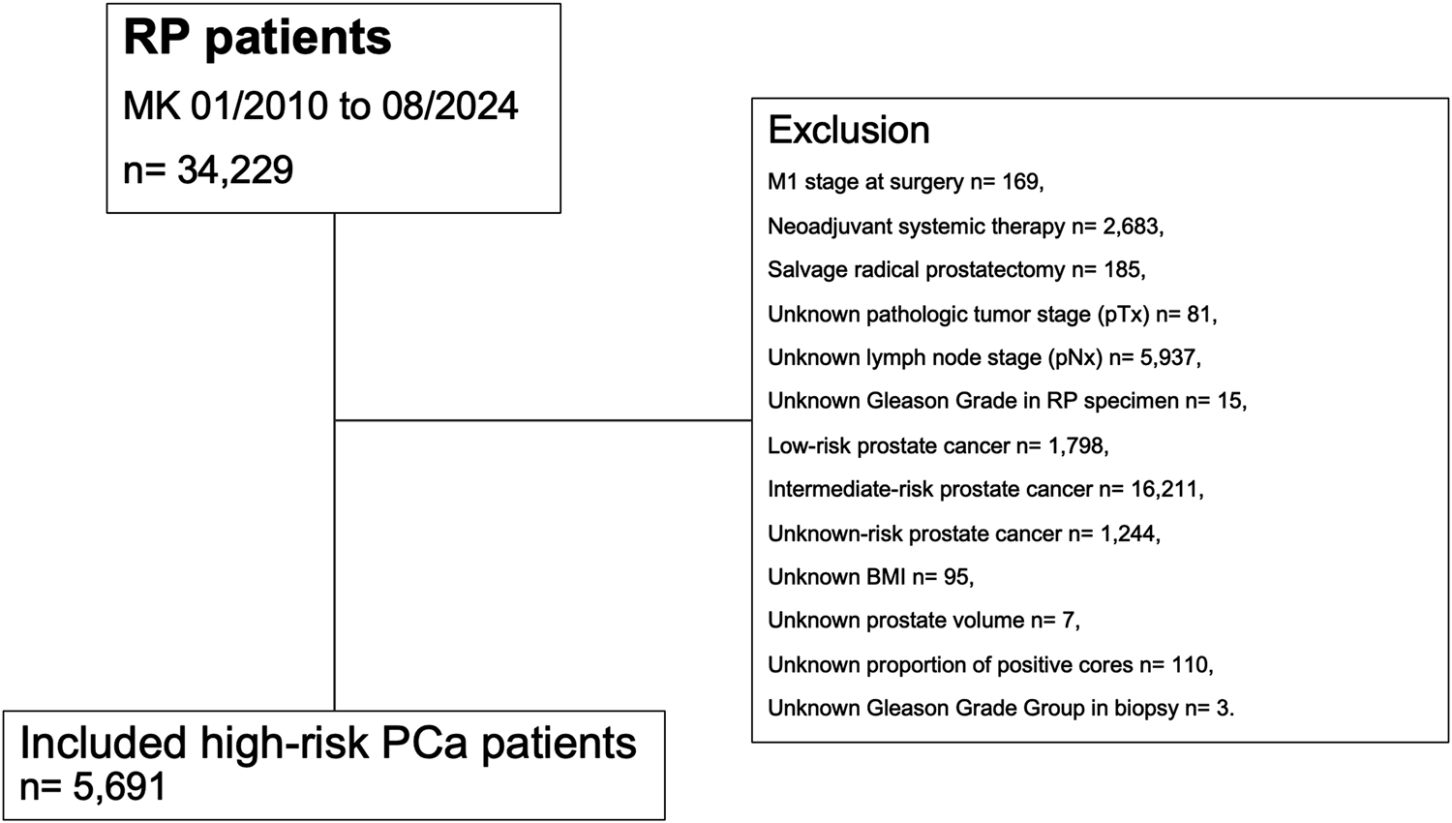
Consort diagram. BMI body mass index, MK Martini Klinik, PCa prostate cancer, pN pathologic lymph node stage at surgery, pT pathologic tumor stage at surgery, RP radical prostatectomy.

After approval by our institutional review board, we retrospectively identified D’Amico high-risk prostate cancer patients who were treated with radical prostatectomy at the high-volume tertiary care center, namely Martini-Klinik (Hamburg, Germany), between January 2010 and August 2024. All patients with clinical suspicion of metastases at time of surgery (cM1), neoadjuvant systemic therapy (chemotherapy and/or hormonal therapy), and previous radiation therapy of the prostate (salvage radical prostatectomy) were excluded. Only patients with complete data regarding tumor stage, lymph node stage, Gleason Grade Group, body mass-index (BMI), prostate volume, and proportion of positive biopsy cores were included in the study cohort (Fig. 1).

All clinical and patient data were stored in a secured and pseudonymized prospectively maintained database. All analyzed patients gave their informed written consent for data collection and analysis. Approval by the local ethics committee has been obtained for data collection. Reporting follows the precepts established by the Helsinki Declaration.

### Definition of endpoint and variables of interest

The primary endpoint of the study consisted of predicting the indication for adjuvant radiation therapy after radical prostatectomy according to current guideline recommendations of the EAU [7, 8]. For each patient, the following preoperative covariates were recorded: age at surgery (continuously, in years), BMI (continuously, in kg/m^2^), prostate volume (> 45 vs. 25–45 vs. <25 cm^3^), prostate-specific antigen (PSA) value (>20 vs. 10–20 vs. <10 ng/ml), clinical tumor stage based on digital rectal examination (cTstage: cTx vs. cT3/cT4 vs. cT2 vs. cT1), number of positive cores at biopsy (> 12 vs. ≤12), proportion of positive cores in biopsy (continuously, in %), highest Gleason Grade Group per core in biopsy (5 vs. 4 vs. 3 vs. 2 vs. 1).

### Statistical analyses

First, preoperative patient and clinical tumor characteristics were tabulated according to the indication for adjuvant radiation therapy. For continuously coded variables, medians and interquartile ranges (IQR) were reported. For categorical variables, frequencies and respective proportions were recorded. Second, the distributions of pathologic tumor stage, pathologic lymph node stage, surgical margin status, and Gleason Grade Group in radical prostatectomy specimen were tabulated. Third, univariable logistic regression models addressed the indication for adjuvant radiation therapy. Subsequently, only risk factors that achieved statistical significance (*p* < 0.05) in univariable logistic regression models were included in multivariable logistic regression models. The accuracy of predicting the indication for adjuvant radiation therapy for all individual variables as well as their combination were quantified using Harrel’s concordance index (c-index) [14]. Step-wise regression method with comparison of c-indexes of each model was used to identify the most accurate multivariable model. The predictive accuracy of multivariable models was internally validated using 2000 bootstrap resamples [15]. Additionally, the corresponding 95% confidence intervals were estimated. Finally, to illustrate the effect of each included risk factor within the multivariable model and to quantify the individual risk of a patient prior to radical prostatectomy, a nomogram was plotted.

R software environment was used for statistical computing and graphics (R version 4.3.2; R Foundation for Statistical Computing, Vienna, Austria) [16]. All tests were two sided, with a significance level set at *p* < 0.05.

## Results

### Clinical characteristics

Within our high-volume center database, 5691 high-risk prostate cancer patients treated with radical prostatectomy between 01/2010 and 08/2024 met the above-described inclusion criteria (Fig. 1). Median age at surgery was 66 years (IQR 60–70 years), median BMI was 26.5 kg/m^2^ (IQR 24.6–29.1 kg/m^2^), median prostate volume was 32 cm^3^ (IQR 25–45 cm^3^), and median preoperative PSA-value was 10.2 ng/ml (IQR 6.3–22.3 ng/ml; Table 1). Addressing clinical tumor characteristics, most patients exhibited cT2 stage (69%), had ≤12 sampled biopsy cores (77%), and harbored Gleason Grade Group 4 in biopsy (50%). Median proportion of positive cores in biopsy was 50% (IQR 30-70%).

**Table 1:** Clinical characteristics of 5691 high-risk prostate cancer patients treated with radical prostatectomy between 01/2010 and 08/2024 at a high-volume center.

Characteristic		Overall, *n* = 5691	Indication for adjuvant radiation therapy, *n* = 2137 (38%)^a^	No indication for adjuvant radiation therapy, *n* = 3554 (62%)^a^	*p* value^b^
**Age at surgery** (in years)		66 (60, 70)	66 (61, 70)	66 (60, 70)	0.6
**BMI** (in kg/m^2^)		26.5 (24.6, 29.1)	26.6 (24.6, 29.1)	26.5 (24.6, 29.0)	0.3
**Prostate volume** (in cm^3^)		32 (25, 45)	35 (26, 48)	30 (23, 42)	**<0.001**
	<25	1393 (24%)	383 (18%)	1010 (28%)	**<0.001**
	25–45	3006 (53%)	1172 (55%)	1834 (52%)	
	>45	1292 (23%)	582 (27%)	710 (20%)	
**PSA** (in ng/ml)		10.2 (6.3, 22.3)	12.5 (7.4, 24.8)	9.1 (5.9, 21.1)	**<0.001**
	<10	2777 (49%)	853 (40%)	1924 (54%)	**<0.001**
	10–20	1168 (21%)	554 (26%)	614 (17%)	
	>20	1746 (31%)	730 (34%)	1016 (29%)	
**cTstage**	cT1	1376 (24%)	318 (15%)	1058 (30%)	**<0.001**
	cT2	3896 (69%)	1605 (75%)	2291 (65%)	
	cT3/cT4	240 (4%)	153 (7%)	87 (2%)	
	cTx	179 (3%)	61 (3%)	118 (2%)	
**Number of sampled biopsy cores** ≤ **12**		4376 (77%)	1751 (82%)	2625 (74%)	**<0.001**
**Proportion of positive cores in biopsy** (in %)		50 (30, 70)	62 (42, 90)	42 (25, 60)	**<0.001**
**Gleason Grade Group in biopsy**	1	246 (4%)	37 (2%)	209 (6%)	**<0.001**
	2	540 (10%)	136 (6%)	404 (11%)	
	3	436 (7%)	190 (9%)	246 (7%)	
	4	2833 (50%)	841 (39%)	1992 (56%)	
	5	1636 (29%)	933 (44%)	703 (20%)	
**Robotic-assisted radical prostatectomy**		2549 (45%)	783 (37%)	1766 (50%)	**<0.001**
**Nervesparing**		4801 (86%)	1519 (72%)	3282 (94%)	**<0.001**

*BMI* body mass index, *cTstage* clinical tumor stage, *PSA* prostate-specific antigen.

^a^Median (interquartile range); *n* (%).

^b^Wilcoxon rank sum test; Pearson’s Chi-square test.

### Pathological characteristics

**Fig. 2:**
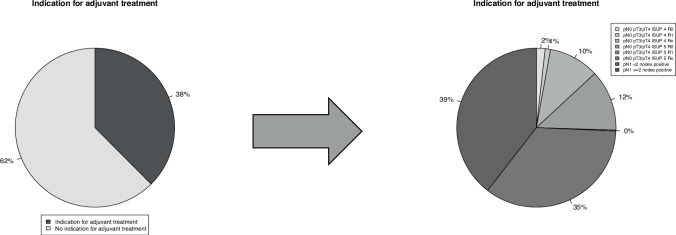
Indication for adjuvant radiation therapy according to the current prostate cancer guidelines of the European Association of Urology (EAU 2025) in 5691 high-risk prostate cancer patients treated with radical prostatectomy between 01/2010 and 08/2024. pN pathologic lymph node stage at surgery, pT pathologic tumor stage at surgery, R surgical margin status at surgery.

In 5691 high-risk prostate cancer patients treated with radical prostatectomy, 3710 (65%) had non-organ confined tumor stage (pT3/pT4), 1588 (28%) had pathological positive lymph nodes (pN1), and 1970 (35%) had positive surgical margins (R1). Distribution of Gleason Grade Group in radical prostatectomy specimen was from lowest (ISUP 1) to highest (ISUP 5) as follow: 1, 32, 42, 2, 23%. These pathological characteristics translated into the indication for adjuvant radiation therapy after radical prostatectomy based on current EAU guideline recommendations for 2137 (38%) patients (Fig. 2).

### Univariable and multivariable logistic regression models addressing the indication for adjuvant radiation therapy

**Fig. 3:**
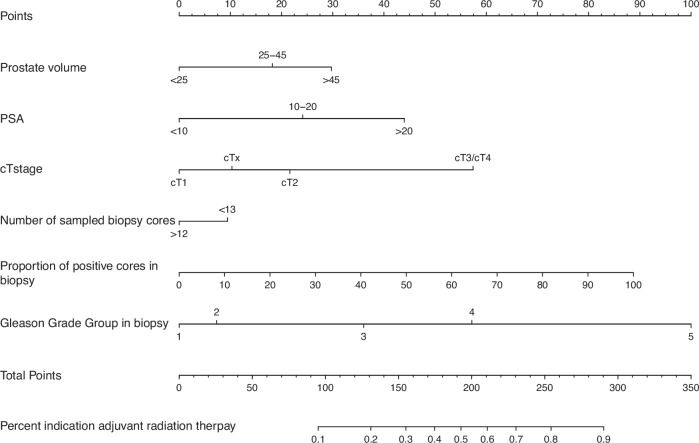
Nomogram addressing the indication for adjuvant radiation therapy after radical prostatectomy according to the current prostate cancer guidelines of the European Association of Urology (EAU 2025) in 5691 high-risk prostate cancer patients treated with radical prostatectomy between 01/2010 and 08/2024. cT clinical tumor stage, PSA prostate-specific antigen.

In univariable logistic regression models predicting the indication for adjuvant radiation therapy, six of eight covariates achieved statistical significance (Table 2). These risk factors were higher prostate volume (> 45 cm^3^: odds ratio [OR] 2.16, 95% confidence interval [CI] 1.84–2.54; and 25–45 cm^3^: OR 1.69, 95% CI 1.47–1.94), higher preoperative PSA-value (>20 ng/ml: OR 1.62, 95% CI 1.43-1.84; and 10–20 ng/ml: OR 2.04, 95% 1.78–2.35), advanced clinical tumor stage (cT3/cT4: 5.85, 95% 4.38–7.86; and cT2: OR 2.33, 95% CI 2.03–2.69), lower number of sampled biopsy cores (≤ 12: OR 0.62, 95% CI 0.55–0.71), higher proportion of positive cores in biopsy (OR 1.026, 95% 1.024–1.028), and higher Gleason Grade Group in biopsy (ISUP 5: OR 7.50, 95% 5.28-10.93; ISUP 4: OR 2.39, 95% 1.69-3.46; ISUP 3: OR 4.36, 95% 2.96–6.57; ISUP 2: OR 1.90, 95% 1.29–2.87). Corresponding c-index accuracy values were 0.568, 0.579, 0.592, 0.540, 0.688, and 0.648, respectively (Table 2). In multivariable logistic regression models, all six included covariates achieved independent predictor status, namely higher prostate volume (> 45 cm^3^: OR 1.95, 95% CI 1.63–2.34; and 25–45 cm^3^: OR 1.51, 95% CI 1.30–1.76), higher preoperative PSA-value (>20 ng/ml: OR 2.69, 95% CI 2.21–3.29; and 10–20 ng/ml: OR 1.72, 95% 1.48–2.01), advanced clinical tumor stage (cT3/cT4: 3.64, 95% 2.58–5.16; and cT2: OR 1.63, 95% CI 1.40–1.90), lower number of sampled biopsy cores (> 12: OR 0.81, 95% CI 0.70-0.9), higher proportion of positive cores in biopsy (OR 1.020, 95% 1.018–1.023), and higher Gleason Grade Group in biopsy (ISUP 5: OR 9.50, 95% 6.34–14.53; ISUP 4: OR 3.62, 95% 2.43–5.51; ISUP 3: OR 2.25, 95% 1.50–3.46). The c-index accuracy of predicting the indication for adjuvant radiation therapy based on preoperative patient and clinical tumor characteristics was 0.761 (95% CI 0.749–0.776) after 2000 bootstrap resamples for internal validation. The multivariable model was graphically displayed using the nomogram format where Gleason Grade Group represented the most influential risk factor for the indication for adjuvant radiation therapy after radical prostatectomy (Fig. 3).

**Table 2:** Univariable and multivariable logistic regression models addressing the indication for adjuvant radiation therapy after radical prostatectomy according to the current prostate cancer guidelines of the European Association of Urology (EAU 2025) in 5691 high-risk prostate cancer patients treated with radical prostatectomy between 01/2010 and 08/2024.

	Univariable				Multivariable*			
	OR	95% CI	*p* value	c-index	OR	95% CI	*p* value	c-index
**Age at surgery** (continuous)	1.002	0.994, 1.01	0.6	0.505	—	—		0.761**
**BMI** (continuous)	1.01	0.994, 1.023	0.2	0.508	—	—		
**Prostate volume** (Ref. <25 cm^3^)	—	—		**0.568**	—	—		
25-45 cm^3^	**1.69**	1.47, 1.94	**<0.001**		**1.51**	1.30, 1.76	**<0.001**	
>45 cm^3^	**2.16**	1.84, 2.54	**<0.001**		**1.95**	1.63, 2.34	**<0.001**	
**PSA** ( < 10 ng/ml)	—	—		**0.579**	—	—		
10–20 ng/ml	**2.04**	1.78, 2.35	**<0.001**		**1.72**	1.48, 2.01	**<0.001**	
>20 ng/ml	**1.62**	1.43, 1.84	**<0.001**		**2.69**	2.21, 3.29	**<0.001**	
**cTstage** (Ref. cT1)	—	—		**0.592**	—	—		
cT2	**2.33**	2.03, 2.69	**<0.001**		**1.63**	1.40, 1.90	**<0.001**	
cT3/cT4	**5.85**	4.38, 7.86	**<0.001**		**3.64**	2.58, 5.16	**<0.001**	
cTx	**1.72**	1.23, 2.39	**0.001**		1.26	0.87, 1.81	0.2	
**Number of sampled biopsy cores** > **12** (Ref. ≤12)	**0.62**	0.55, 0.71	**<0.001**	**0.540**	**0.81**	0.70, 0.94	**0.005**	
**Proportion of positive cores in biopsy** (continuous)	**1.026**	1.024, 1.028	**<0.001**	**0.688**	**1.020**	1.018, 1.023	**<0.001**	
**Gleason Grade Group in biopsy** (Ref. ISUP 1)	—	—		**0.648**	—	—		
2	**1.90**	1.29, 2.87	**0.002**		1.18	0.78, 1.81	0.4	
3	**4.36**	2.96, 6.57	**<0.001**		**2.25**	1.50, 3.46	**<0.001**	
4	**2.39**	1.69, 3.46	**<0.001**		**3.62**	2.43, 5.51	**<0.001**	
5	**7.50**	5.28, 10.93	**<0.001**		**9.50**	6.34, 14.53	**<0.001**	

*adjusted for prostate volume, PSA value, cTstage, number of sampled biopsy cores, proportion of positive cores in biopsy, Gleason Grade Group in biopsy.

**after 2000 bootstrap resamples for internal validation, 95% confidence interval: 0.749–0.776.

*BMI* body mass index, *CI* confidence interval, *cTstage* clinical tumor stage, *OR* odds ratio, *PSA* prostate-specific antigen.

For each variable, c-indices evaluate the concordance between predicted and observed indication for adjuvant radiation therapy after radical prostatectomy.

## Discussion

Following radical prostatectomy, some patients will have adverse pathologic features and positive lymph nodes. While the treatment guidelines of the AUA and the NCCN adopt a cautious approach toward recommending adjuvant therapy following radical prostatectomy [9, 10], current EAU guidelines recommend adjuvant radiation therapy in patients with pathological negative lymph node stage (pN0) with Gleason Grade Group 4–5 and non-organ confined pathological tumor stage (pT3) with or without positive surgical margins as well as in patients with pathological positive lymph node stage [7, 8]. Within the current study, we hypothesized that patient and clinical tumor characteristics may predict these indications for adjuvant radiation therapy in high-risk prostate cancer patients. We tested this hypothesis according to standard testing criteria and made several noteworthy observations.

First, in our contemporary cohort of 5691 high-risk prostate cancer patients treated with radical prostatectomy at a high-volume center, 65% exhibited non-organ confined tumor stage and 35% had positive surgical margins. These distributions of pathological tumor characteristics are comparable to other contemporary analyses addressing radical prostatectomy specimen of patients with preoperatively high-risk classified prostate cancer in other tertiary-care centers in North America, Europe, and Asia [17–20]. Conversely, rates of pathological positive lymph nodes as high as 28% reported within the present study are higher than those previously reported, ranging from 8 to 24% [17–20]. These differences in the rates of positive lymph nodes may be explained by differences in the extension of pelvic lymph node dissection, and therefore, higher number of sampled lymph nodes within our institution. In addition, specialized uropathologists at our institution completing comprehensive preparation of the specimen may contribute to a higher lymph node count compared to other institutions.

Second, based on current EAU guideline recommendations, the pathological characteristics, reported within the present study cohort, translated into indications for adjuvant radiation therapy after radical prostatectomy for 38% of patients. Conversely, in the real world, only 3% of patients with adverse pathology outcomes received adjuvant radiation therapy within six months after radical prostatectomy [5]. Instead, the larger proportion of patients (18%) underwent early salvage radiation therapy at a median PSA-value of 0.3 ng/ml [5]. The RADICALS-RT, RAVES, and GETUC-AFU 17 trials, all prospective randomized controlled trials published in 2020, found no significant difference in biochemical progression-free survival between adjuvant and early salvage radiotherapy [21–23]. Similarly, the ARTISTIC meta-analysis of these three trials reported no difference in event-free survival (pooled hazard ratio 0.95; 95% CI 0.75–1.21) [24]. Based on these observations, the AUA and NCCN guidelines are cautious regarding the role of adjuvant radiotherapy [9, 10]. However, the proportion of patients with adverse pathologic features at radical prostatectomy (pT3, Gleason Grade Group 4–5 with or without positive surgical margins) was low in RADICALS-RT, RAVES, and GETUC-AFU 17 trial [21–23]. Therefore, even the ARTISTIC meta-analysis may have been underpowered to show a difference in treatment outcomes [8, 24]. In contrast to these findings, Tilki et al. demonstrated within two retrospective multicenter studies that adjuvant radiation therapy is associated with more favorable overall survival compared to early salvage radiation according to the EAU guidelines in patients with pathologic positive lymph nodes (pN1) or pathologic Gleason Grade Group 4–5 and pT3/4 stage [5, 6, 8]. Moreover, Knipper et al. reported that patients with non-organ confined (pT3) prostate cancer and either positive surgical margins (R1) and/or positive lymph nodes (pN1) following multidisciplinary adjuvant radiation therapy recommendations were less likely to experience biochemical recurrence, metastatic progression, cancer-specific mortality, as well as overall mortality [25]. These considerations validate the need for the present study, developing a multivariable model, that may be used in preoperative patient counseling to access the risk for the indication for adjuvant radiation therapy.

Third, in the current study, preoperatively known baseline characteristics of patients with indication for adjuvant radiation therapy differed from those of their counterparts without indication for adjuvant radiation therapy. Specifically, in patients with indication for adjuvant radiation therapy, median prostate size was larger (35 vs. 30 cm^3^), median preoperative PSA-value was higher (12.5 vs. 9.1 ng/ml), and clinical suspicious tumor stage (cT3/cT4: 7 vs. 2% and cT2: 75 vs. 65%) was more frequent. Moreover, the proportion of patients with ≤12 sampled cores in biopsy (82 vs. 74%) as well as the median proportion of positive cores in biopsy (62 vs. 42%) was higher, and Gleason Grade Group 5 (44 vs. 20%) was more frequent. These differences validate the rational of our hypotheses that patient and clinical tumor characteristics may predict indication for adjuvant radiation therapy in high-risk prostate cancer patients.

Fourth, in subsequently completed multivariable regression models, six preoperative known patient and clinical tumor characteristics achieved independent predictor status, namely prostate volume (> 45 cm^3^ and 25–45 cm^3^), preoperative PSA-value (>20 ng/ml and 10–20 ng/ml), clinical tumor stage (cT3/cT4 and cT2), number of sampled biopsy cores (≤ 12), proportion of positive cores in biopsy, and Gleason Grade Group in biopsy (ISUP 5, ISUP 4 and ISUP 3). The c-index accuracy of predicting the indication for adjuvant radiation therapy was 0.761 (95% CI 0.749–0.776) after 2000 bootstrap resamples for internal validation. To the best of our knowledge, we are the first to report such multivariable model addressing the indication and quantifying the need of adjuvant radiation therapy based on current EAU guideline recommendations. In consequence, the multivariable model described within the present study cannot be compared to any previous model. Nevertheless, the currently presented model includes variables such as PSA-value, Gleason Grade Group in biopsy, clinical tumor stage, and biopsy core involvement—features also used in the well-established nomogram of Memorial Sloan Kettering Cancer Center (MSKCC) for the prediction of lymph node involvement, seminal vesicle invasion, or organ-confined disease [26, 27]. This overlap supports the clinical relevance of our model inputs and facilitates comparison with existing predictive tools, though differences in endpoints and patient cohorts warrant further validation.

Taken together, 2137 of 5691 (38%) high-risk prostate cancer patients had the indication for adjuvant radiation therapy after radical prostatectomy based on current EAU guideline recommendations. Indication for adjuvant radiation therapy was associated with higher prostate volume, higher PSA-value, advanced tumor stage, higher proportion of positive cores, and higher Gleason Grade Group in biopsy. Conversely, indication for adjuvant radiation therapy was predicted by lower number of sampled biopsy cores. The multivariable model, proposed within the current study, exhibited a c-index accuracy value of 0.761 for prediction of the indication for adjuvant radiation therapy. The c-index accuracy value of the suggested multivariable model was superior to the usual cut-off of 0.700, which should be exceeded by a model to warrant consideration for prediction in individual patients. Therefore, the above-described multivariable model may be of great value for patients and clinicians to better anticipate and quantify the risk for adjuvant treatment after radical prostatectomy. Moreover, the multivariable model may provide both urologists as well as radio oncologists significant support in patient counseling regarding alternative curative therapy options for high-risk non-metastatic prostate cancer prior treatment decision-making to reduce post-treatment decision regret.

The current study is not devoid of limitations. First and foremost, it shares the same constrains as all other retrospective single-center studies [18, 28–30]. Second, since multiparametric magnetic resonance imaging (mpMRI) of the prostate is not covered by the German public health insurance, mpMRI-associated variables such as number, location, and degree of PIRADS lesions were only available for a minority of patients and could therefore not be considered in the development of the present multivariable model. Additionally, detailed information regarding the proportion of Gleason 4 pattern in biopsy as well as maximum percentage of tumor in biopsy cores are unknown for the majority of patients. Third, documented post-prostatectomy radiation therapy rates are low within the present database. In consequence, the rates of guideline-based indications for adjuvant radiation therapy were not compared to actual adjuvant and early-salvage radiation therapy rates. Last but not least, the currently described multivariable model demonstrates promising predictive performance within our cohort. However, its generalizability to other patient populations and clinical settings remains uncertain. Therefore, external validation in independent cohorts is necessary to confirm its accuracy, calibration, and clinical utility before it can be recommended for broader use in clinical practice. Despite these limitations, the current study demonstrates the effectiveness of a multivariable model predicting the indication and quantifying the need of adjuvant radiation therapy based on patient and clinical tumor characteristics known prior to radical prostatectomy.

## Conclusions

Clinical tumor characteristics can be used for preoperatively predicting the indication and quantifying the need for adjuvant radiation therapy after radical prostatectomy according to current EAU guideline recommendations in high-risk prostate cancer patients. However, the currently presented multivariable model, developed at a single-institution, should be externally validated in an independent cohort to ensure its reliability and generalizability prior to broader clinical application.

## Data Availability

All data generated or analyzed during this study are included in this article. Further enquiries can be directed to the corresponding author.
